# Targeting IL8 as a sequential therapy strategy to overcome chemotherapy resistance in advanced gastric cancer

**DOI:** 10.1038/s41420-022-01033-1

**Published:** 2022-04-29

**Authors:** Huning Jiang, Jiahua Cui, Hao Chu, Tingting Xu, Mengyan Xie, Xinming Jing, Jiali Xu, Jianwei Zhou, Yongqian Shu

**Affiliations:** 1 Department of Oncology, The First Affiliated Hospital of Nanjing Medical University, Nanjing, China; 2 Jiangsu Key Lab of Cancer Biomarkers, Prevention and Treatment, Collaborative Innovation Center for Cancer Personalized Medicine, Nanjing Medical University, Nanjing, China; 3 Department of Molecular Cell Biology & Toxicology, Center for Global Health, School of Public Health, Nanjing Medical University, Nanjing, China; 4 Department of Oncology, Sir Run Run Hospital, Nanjing Medical University, Nanjing, China

**Keywords:** Gastric cancer, Chemokines, Targeted therapies

## Abstract

Systemic chemotherapy with multiple drug regimens is the main therapy option for advanced gastric cancer (GC) patients. However, many patients develop relapse soon. Here, we evaluated the therapeutic potential of targeting interleukin-8 (IL8) to overcome resistance to chemotherapy in advanced GC. RNA sequencing revealed crucial molecular changes after chemotherapy resistance, in which the expression of IL8 was significantly activated with the increase in drug resistance. Subsequently, the clinical significance of IL8 expression was determined in GC population specimens. IL8-targeted by RNA interference or reparixin reversed chemotherapy resistance with limited toxicity in vivo and vitro experiments. Sequential treatment with first-line, second-line chemotherapy and reparixin inhibited GC growth, reduced toxicity and prolonged survival. Collectively, our study provides a therapeutic strategy that targeting IL8 as a sequential therapy after chemotherapy resistance in advanced GC.

## Introduction

GC is the fifth most common cancer and the third leading cause of cancer-related death in the world [1, 2]. To date, systemic chemotherapy with multiple drug regimens is the main therapy option for patients with recurrent and metastatic GC [3]. Advanced GC is treated with sequential lines of chemotherapy, starting with a platinum and fluoropyrimidine doublet as the first-line, and continuing with taxane or irinotecan in the second-line setting [4–6]. However, many patients develop relapse soon because tumor heterogeneity facilitates the escape from cytotoxic therapies [7]. Treatment-induced upregulation of genes including those associated with multi-drug resistance (MDR) enhances chemotherapeutic resistance in cancer cells [8]. Moreover, the serious toxic effects such as myelosuppression, oral or gastrointestinal toxicity, neuropathy, thromboembolic disease and renal dysfunction may cause patients to discontinue or reduce chemo-drugs [9, 10]. Therefore, identifying well-tolerated therapeutic drugs and reducing systemic toxic effects may be the best strategy for prolonging the survival of advanced GC patients.

Accurately identifying drug-resistance targets after chemotherapy and implementing sequential therapy may effectively reverse drug resistance [11]. In addition, the multi-organ toxic effects caused by long-term chemotherapy may be avoided. It has been reported that neoadjuvant atezolizumab in combination with sequential nab-paclitaxel and anthracycline-based chemotherapy significantly improved pathological complete response rates with an acceptable safety profile [12]. Luminal B-like breast cancer with a basal molecular subtype and a state of immune activation may respond to sequential anthracyclines and anti-PD-1 [13]. Hence, we speculated that after first-line and second-line chemotherapy for advanced GC, screening drug-resistance targets and implementing sequential therapy may effectively inhibit tumors and limit toxic effects.

Cytokines act as crucial mediators of cell communication in tumor microenvironment (TME), as well as important therapeutic targets and prognostic factors [14]. Tumor cells can acquire the expression of various cytokines and their receptors to exploit these molecules to aid in the growth, survival, and spread of the tumor. Tumor cells may also benefit directly from cytokine signaling if they have gained the expression of the cognate cytokine receptors, thereby allowing them to activate autocrine positive feedback loops. One such cytokine/receptor pair is the interleukin (IL)8/IL8R. IL8, alternatively known as CXCL8, is a pro-inflammatory chemokine whose function is mediated by binding to cell-surface G protein-coupled receptors, termed CXCR1 and CXCR2 [15]. IL8 is produced by many cell types including endothelial cells, macrophages, epithelial cells, monocytes, and fibroblasts. In unstimulated cells, IL8 levels are almost undetectable, which increases by 10–100 folds, in response to a variety of factors including cytokines (IL-1, IL-6, CXCL12, and TNF-α), hypoxia, reactive oxygen species (ROS), pathogen-associated molecular patterns (PAMPs), and other environmental stressors [16]. High expression levels of IL8 have been observed in various cancers [17–22].

The increased synthesis and secretion of IL8 has wide effects on TME given the characterized expression of CXCR1 and CXCR2 receptors on cancer cells, endothelial cells, neutrophils and tumor-associated macrophages(TAMs) [23]. The IL8-CXCR1/2 axis plays an important role in tumor growth, angiogenesis, metastasis, stemness, and recruitment of immune cells into the TME, by activation of downstream signaling cascades such as the PLC-PKC, PI3K-AKT, FAK/Src, MAPK-ERK, and RhoGTPase [24, 25]. This cytokine signaling can substantially alter leukocyte infiltration into the tumor, resulting in the accumulation of immunosuppressive and pro-tumorigenic immune cells that can provoke the dysfunction of cytotoxic antitumor immune cells [25]. The IL8-CXCR1/2 axis can modulate the phenotypic status of tumor cells by activating a cellular differentiation program known as epithelial–mesenchymal transition (EMT), which endows tumor cells with enhanced metastatic, stemness, and resistance qualities [26].

Owing to the crucial functions of the IL8-CXCR1/2 signaling in cancer, targeted against IL8 is expected to have high clinical value in tumor therapy. Reparixin, a non-competitive allosteric inhibitor of CXCR1/2, was shown to prevent the activated receptor-induced intracellular signal transduction cascade and cell response by locking CXCR1 and CXCR2 in an inactive conformation [27]. Reparixin is a potent functional inhibitor of IL8-induced biological activities in human leukocytes by potently and selectively blocking human polymorphonuclear leukocytes (PMN) adhesion, PMN activation including release of granule content and pro-inflammatory cytokine production as well as T lymphocyte and NK cell migration induced by IL8 [27]. Preclinical studies have shown that reparixin was able to specifically target the CSC population in human breast cancer xenografts, retarding tumor growth and reducing metastasis. Moreover, several trials have substantiated the synergistic functions of reparixin in combination with chemotherapeutic agents [28–30]. These studies have substantiated the potential for IL8 inhibition to be utilized as a single agent or in combination with chemotherapeutic agents. However, little evidence is available concerning its potential as a targeted agent after resistance to first-line and second-line chemotherapy in advanced GC.

In this study, we clarified the effects of IL8-targeted therapy after first-line and second-line chemotherapy in advanced GC. Moreover, we further evaluated the toxic effects on a human gastric cancer cell (BGC823) and a human gastric mucosal cell (GES-1) after sequential treatment with first-line, second-line chemotherapy and reparixin. This work provides a blueprint for exploring the feasibility of targeting IL8 as a sequential therapeutic strategy after chemotherapy resistance.

## Results

### First-line chemotherapy effectively inhibits GC growth but develops drug resistance

**Fig. 1:**
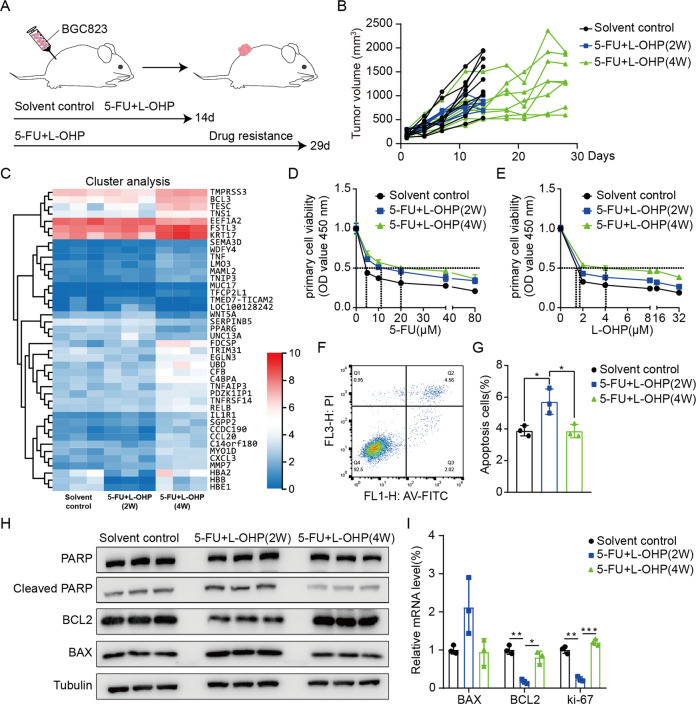
First-line chemotherapy effectively inhibits GC growth but develops drug resistance. **A** Schematic representation of the BGC823-bearing model of first-line chemotherapy resistance. **B** The growth curves of tumor (*n* = 8 per group). **C** Hierarchical clustering gene transcription altered at different periods of chemotherapy. **D**, **E** The half-maximal inhibitory concentration (IC50) was measured by CCK8 assay. Primary cells from dissected tumor tissues were treated with different concentrations of 5-Fu (0, 5, 10, 20, 40, and 80 μM) and oxaliplatin (0, 2, 4, 8, 16 and 32 μM). **F**, **G** Flow cytometric assay of primary cells from dissected tumor tissues. Data are mean ± standard deviation. *P < 0.05. **H** Protein expression of apoptosis-related proteins (PARP, cleaved PARP, Bcl2, and Bax) were detected by western blot analysis in dissected tumor tissues. **I** The mRNA expression of Bax, Bcl2, and ki-67 in dissected tumor tissues were detected by qRT-PCR assay. Data are mean ± standard deviation. *P < 0.05, **P < 0.01, ***P < 0.001.

According to NCCN guidelines [6], we chose 5-Fu and oxaliplatin as the first-line chemotherapy regimen for GC. As shown in Fig. 1A, we constructed the xenograft mouse model by BGC823 cells and found that 5-Fu and oxaliplatin showed obvious anti-proliferation activity compared with solvent treatment at the first 2 weeks (Figs. 1B and S1A, B). Moreover, the tumor growth rapidly increased on the 25th day, while body weight continuously decreased (Figs. 1B and S1C), indicating chemotherapeutic resistance may occur after 4 weeks of injection. RNA-seq analysis of tumor tissues revealed genetic changes at different periods of chemotherapy (Fig. 1C). Compared with solvent treatment, expression of some oncogenes (such as TMPRSS3, BCL3, TESC, etc.) declined after 2 weeks of chemotherapy and then resumed expression after 4 weeks of chemotherapy, achieving levels that were even higher than in the control group. KEGG pathway analysis also showed that compared with 2 weeks of chemotherapy, some tumor-related signaling pathways were enriched after 4 weeks of chemotherapy, and the most enriched pathway was NF-κB signaling pathway (Fig. S1D).

To further confirm whether the tumor cells developed drug resistance after 4 weeks of chemotherapy, we detected chemosensitivity of primary cells extracted from tumor tissues. The IC50 of primary cells after 4 weeks of chemotherapy-treated with 5-Fu and oxaliplatin was higher than that of primary cells after 2 weeks of chemotherapy (Fig. 1D, E). Flow cytometry analysis showed that the apoptosis of cells after 4 weeks of treatment was lower than in cells after 2 weeks of treatment (Fig. 1F, G). Furthermore, the expression of proliferation and apoptosis-related genes were examined by western blotting and quantitative polymerase chain reaction (qRT-PCR) assays. The results exhibited that Bcl-2 and ki-67 were increased in primary cells after 4 weeks of chemotherapy, and the expression of cleaved PARP and Bax were decreased (Figs. 1H, I and S1E), indicating that tumor proliferation was accelerated and apoptosis rate was decreased after 4 weeks of chemotherapy. These results suggest the emergence of chemotherapeutic resistance after 4 weeks of treatment.

### Second-line chemotherapy reverses resistance to first-line chemotherapy of GC

**Fig. 2:**
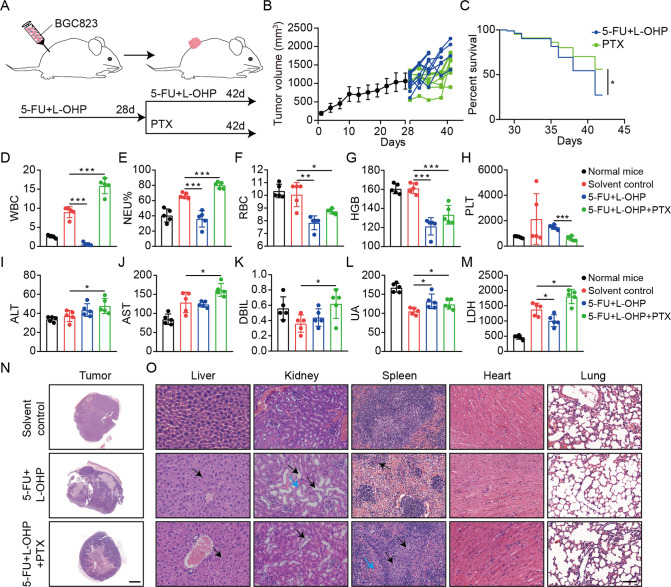
Second-line chemotherapy reverses resistance to first-line chemotherapy of GC. **A** Schematic representation of the BGC823-bearing model of sequential chemotherapy resistance. **B** The growth curves of tumor (*n* = 12 per group). **C** Kaplan–Meier survival curve after 5-Fu plus oxaliplatin or paclitaxel treatments. *P < 0.05. **D**–**H** Blood routine index (WBC, NEU%, RBC, HGB, and PLT) were measured. Data are mean ± standard deviation. *P < 0.05, **P < 0.01, ***P < 0.001. **I**–**M** Blood biochemical index (ALT, AST, DBIL, UA, and LDH) were measured. Data are mean ± standard deviation. *P < 0.05. **N**, **O** The representative images of tumor, liver, kidney, spleen, heart and lung by H&E staining. **N** Scale bars, 2000 μm, **O** Scale bars, 50 μm.

After 5-Fu and oxaliplatin resistance were determined, we chose paclitaxel monotherapy as the second-line chemotherapy regimen [2]. The specific scheme was shown in Fig. 2A. Compared with treatment with 5-Fu and oxaliplatin, the tumor volume was reduced to a certain extent after treatment with paclitaxel, and the survival time of mice was significantly improved (Figs. 2B, C and S2A, B). Moreover, the mice treated with 5-Fu and oxaliplatin lost body weight continuously, while paclitaxel group gradually stabilized (Fig. S2A). These findings suggest paclitaxel is effective after resistance to first-line chemotherapy regimen in GC.

Regardless of the first-line or second-line chemotherapy, mice were found to die under the restriction of tumor growth, suggesting toxic death. To investigate the potential damage to the organs of mice after the first-line or second-line chemotherapy, we performed blood routine examination, blood biochemical analysis and pathological analysis on the organs of mice. Blood routine examination revealed that 5-Fu and oxaliplatin caused severe hematotoxicity characterized by a significant decrease in various blood cells and hemoglobin (HGB) compared with normal mice and solvent control group. Paclitaxel led to a marked increase in white blood cells (WBC), especially neutrophils (NEU), while red blood cells (RBC), HGB and platelets (PLT) were still at low levels (Figs. 2D–H and S3). We speculated that the possibility of infection significantly increased due to decreased body resistance caused by chemotherapy. Blood biochemical analysis showed elevated levels of alanine aminotransferase (ALT), aspartate aminotransferase (AST) and direct bilirubin (DBIL), indicating liver damage (Fig. 2I–K). The increased level of uric acid (UA) reflected that chemotherapy caused a certain degree of kidney injury (Fig. 2L), while the elevated level of lactate dehydrogenase (LDH) after paclitaxel therapy suggested a poor prognosis (Fig. 2M and Fig. S4). Additionally, HE staining showed tumor necrosis foci after first-line and second-line chemotherapy (Fig. 2N). Degenerations of numerous hepatocytes and renal tubules occurred in the chemotherapy group. After chemotherapy, the white pulps of spleen were severely damaged and reduced with a large number of lymphocytes, and the red pulp had numerous extramedullary hematopoietic foci. Heart and lung did not show obvious injuries after chemotherapy (Fig. 2O). The data demonstrated that the toxic effects of chemotherapy were severe and could not be ignored. In the sequential chemotherapy mouse model, first-line and second-line chemotherapy agents led to toxic death under the restriction of tumor growth. With the increase of chemotherapy cycles, the toxicity caused by chemotherapeutic drugs was gradually accumulated, which may lead to irreversible damage to the body. Considering that the adverse reactions caused by the combined chemotherapy strategy may be more serious, it is more reasonable to find new targeted drugs to implement sequential strategy after chemotherapy resistance, which can suppress tumor progression whilst minimizing toxicity.

### Screening IL8 as the therapeutic target after chemotherapy resistance

**Fig. 3:**
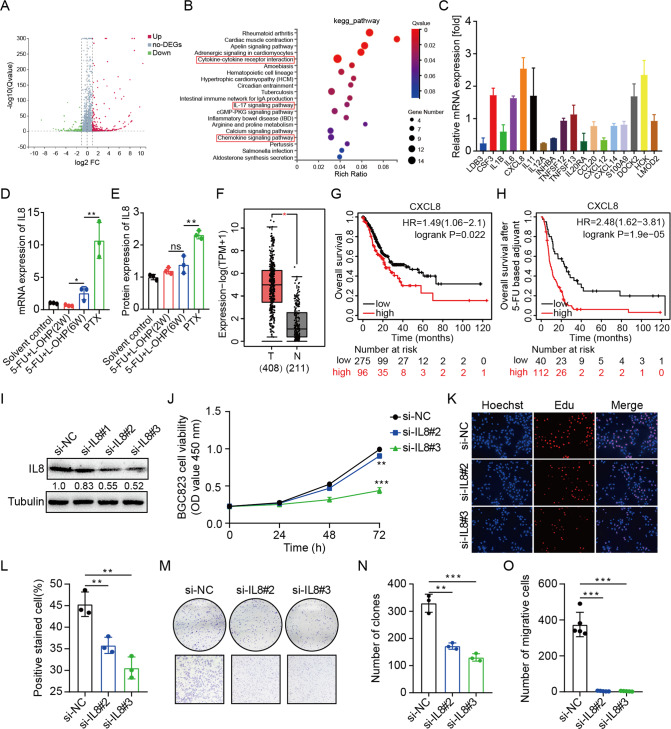
Screening IL8 as the therapeutic target after chemotherapy resistance. **A** The volcano plot of RNA transcription sequencing of the first-line chemotherapy group and the sequential chemotherapy group. A total of 256 differentially expressed genes (DEGs) with fold-change greater than 2. **B** KEGG pathways enriched by DEGs in the transcriptome affected by sequential chemotherapy compared with first-line chemotherapy. **C** qRT-PCR assays were performed to detect the changes of the DEGs in the three enriched signaling pathways most associated with cancer in tumor tissues treated with sequential and first-line chemotherapy. **D**, **E** IL8 expression at different periods of chemotherapy was detected by qRT-PCR assays and western blot analysis. Data are mean ± standard deviation. *P < 0.05, **P < 0.01. **F** Upregulation of IL8 in GC samples was obtained in the GEPIA database. **G** Kaplan–Meier OS curves in GC patients according to the expression of IL8. **H** Kaplan–Meier OS curves in GC patients after 5-Fu therapy according to the expression of IL8. **I** Western blot analysis of IL8 expression in BGC823 cells transfected with siRNAs against IL8. **J** CCK8 assays was carried out to detect the viability of BGC823 cells transfected with si-IL8#2 and si-IL8#3. **P < 0.01, ***P < 0.001. **K**, **L** Representative images (**K**) and quantification (**L**) of BGC823 cells transfected with si-IL8#2 and si-IL8#3 by EdU staining assays. Data are mean ± standard deviation. **P < 0.01. **M**–**O** Representative images (**M**) and quantification (**N**, **O**) of BGC823 cells transfected with si-IL8#2 and si-IL8#3 by colony-forming experiments and transwell assays. Data are mean ± standard deviation. **P < 0.01, ***P < 0.001.

In order to gain preferable molecular insight, we performed RNA-sequencing assays using tumor tissues from sequential chemotherapy and first-line chemotherapy. A set of 182 mRNAs showed an increase in abundance of log_2_FC ≥ 1, while sequential chemotherapy also reduced the abundance of 74 genes (log_2_FC ≤ −1) (Fig. 3A). KEGG pathway analysis was performed on differentially expressed genes (DEGs) in the transcriptome and demonstrated that the potential targets were involved in various signaling pathways, where the three pathways most associated with cancer were cytokine-cytokine receptor interaction, IL-17 signaling pathway and chemokine signaling pathway (Fig. 3B). The intersection of the DEGs in the three enriched signaling pathways were IL8 and CCL20 (Table S1). Then, we performed qRT-PCR assays on the DEGs in Table S1, and the results showed that IL8 exhibited the highest expression level in tumor tissues after sequential chemotherapy compared with the first-line chemotherapy resistance group (Fig. 3C). More importantly, the longer of chemotherapy treatment was associated with the higher of IL8 expression, which were confirmed by RNA-seq data (Fig. S5A), as well as qRT-PCR and Western blot (Figs. 3D, E and S5B). The change of IL8 expression indicates the potential role of IL8 in chemotherapy resistance of advanced GC. In addition, the TCGA database showed that IL8 was highly expressed in GC than normal tissue (Fig. 3F). As shown in the Kaplan–Meier survival curve, the overall survival (OS) of GC patients with high IL8 expression was significantly lower than that of GC patients with low IL8 expression (Fig. 3G), especially in GC patients after 5-Fu therapy (Fig. 3H). Furthermore, we analyzed the relationships between IL8 level and clinical factors of GC patients, finding that the overexpressed IL8 was obviously associated with tumor size, TNM stage and lymphatic metastasis (Table 1 and Fig. S5C). These results suggested that IL8 could be used as a biomarker to predict the therapeutic effect and prognosis of GC.

**Table 1:** Correlation between IL8 expression and clinicopathological features of GC (*n* = 48).

Parameter	IL8 (high)	IL8 (low)	*P*-value (*P < 0.05)
Age (years)			0.7555
≤50	8	7	
>50	16	17	
Sex			0.3502
Male	15	18	
Female	9	6	
Tumor size(cm)			0.0051*
<5	12	21	
≥5	12	3	
Differentiation grade			0.0736
Well-moderate	6	12	
Poor-undifferentiation	18	12	
Lauren classification			0.0907
Intestinal	8	12	
Diffuse	12	12	
Mixed	4	0	
TNM stage			0.0002*
I–II	4	17	
III–IV	20	7	
Lymphatic metastasis			0.0015*
Yes	22	12	
No	2	12	

To clarify the effect of IL8 dysregulation on GC, we designed 3 siRNAs targeting IL8 to knock down IL8 expression in GC cells. The result showed that si-IL8#2 and si-IL8#3 significantly inhibited the expression of IL8 on BGC823 cells (Fig. 3I). CCK8 and EdU assays revealed that silencing IL8 remarkably inhibited BGC823 cells proliferation (Fig. 3J–L), which was also confirmed by colony formation assays (Fig. 3M, N). In addition, transwell assay indicated that the suppression of IL8 attenuated the migratory capacity of BGC823 cells (Fig. 3M, O). These results were also confirmed in SGC7901 cells (Fig. S6). In summary, our findings indicate that IL8 functions as an unfavorable factor in GC and may be used as a therapeutic target after chemotherapy resistance in advanced GC.

### Sequential treatment with first-line, second-line chemotherapy, and reparixin preserves efficacy to GC cells and ameliorates toxicity to normal cells

**Fig. 4:**
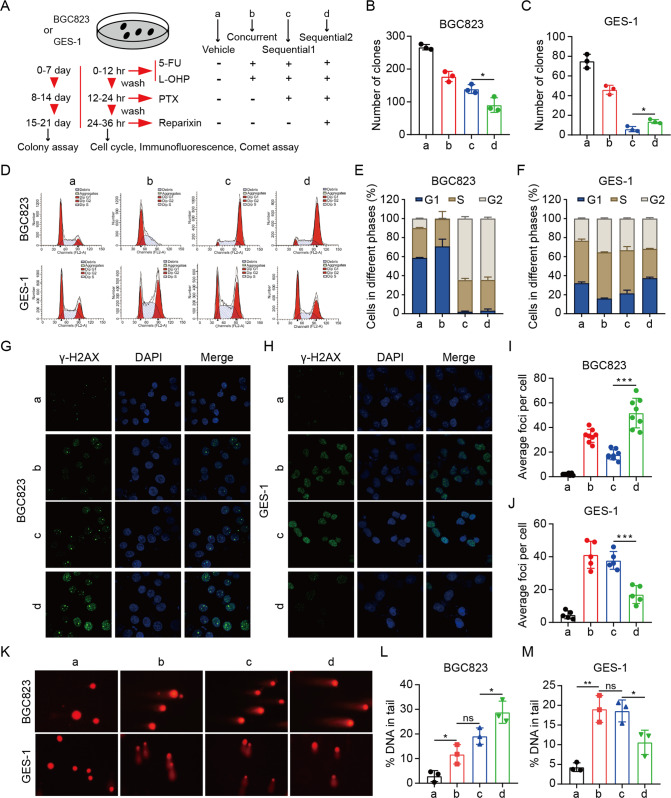
Sequential treatment with first-line, second-line chemotherapy, and reparixin preserves efficacy to GC cells and ameliorates toxicity to normal cells. **A** Schematic representation of sequential treatment with first-line, second-line chemotherapy, and reparixin in BGC823 or GES-1 cells. **B**, **C** Colony formation assays were performed on BGC823 and GES-1 cells treated with different drugs as in Fig. 4A. Data are mean ± standard deviation. *P < 0.05. **D**–**F** BGC823 and GES-1 cells were treated as in Fig. 4A and subjected to flow cytometric analysis. **G**–**J** Representative images (**G**, **H**) and quantification (**I**, **J**) of BGC823 and GES-1 cells treated as in Fig. 4A and then stained for γH2AX and DAPI. Data are mean ± standard deviation. ***P < 0.001. **K**–**M** BGC823 and GES-1 cells were treated as in Fig. 4A and subjected to Comet analysis. DNA damage is quantified as percent DNA in tails. Data are mean ± standard deviation. *P < 0.05, **P < 0.01.

To investigate the effect of targeting IL8 on inhibiting tumor growth, reparixin, a small molecule inhibitor manipulating IL8/IL8R signaling, was selected to restrain the function of IL8. Subsequently, we performed colony, cell cycle, immunofluorescence assay and comet assay of BGC823 cells after sequential treatment with first-line, second-line chemotherapy and reparixin. Furthermore, to further test the toxic effect to normal cells after sequential treatment with first-line, second-line chemotherapy and reparixin, we also performed consistent experiments on GES-1 cells (Fig. 4A). The colony formation assay showed that sequential treatment with reparixin was effective in blocking BGC823 cells growth (Fig. 4B). However, the reduction of colonies caused by the toxicity of sequential chemotherapy was mitigated by reparixin in GES-1 cells (Fig. 4C). Next, we performed flow cytometric analysis to evaluate the effect of sequential treatment on the cell cycle. For BGC823 cells, 5-Fu plus oxaliplatin induced a block of the tumor cells in the G1 phase, while the G2 phase almost completely disappeared. Our results also showed a shift of the tumor cells in the G2 phase after sequential treatment with paclitaxel and reparixin (Fig. 4D, E); however, for GES-1 cells, chemotherapy agents induced G2 arrest was mitigated by reparixin (Fig. 4D, F).

Chemotherapy is an effective therapeutic strategy for GC, mainly eliminating tumors by inducing DNA damage [31]. We performed immunofluorescence assay and comet assay to detect the indicators and extent of DNA damage in tumor cells and normal cells after sequential intervention. Immunofluorescence assay showed that sequential treatment with reparixin led to more γ-H2AX foci in BGC823 cells while it reduced the expression of γ-H2AX in GES-1 cells (Fig. 4G–J). Furthermore, the comet assay demonstrated that sequential treatment with reparixin for BGC823 cells had more and longer tails, indicating the increased DNA damage, whereas the opposite effect was observed in GES-1 cells (Fig. 4K–M). All observations revealed that sequential treatment with first-line, second-line chemotherapy and reparixin preserved efficacy to GC cells and ameliorated toxicity to normal cells. We speculated that endogenous replication stress (RS) in tumor cells, but not in normal cells, underlies the efficacy of sequential reparixin therapy in tumors, while ameliorating toxicity in normal cells. Abnormal DNA damage responses (DDR) and replication stress (RS) result in accumulation of DNA damage contributing to tumor initiation and progression [32–34]. Owing to high endogenous RS in cancer cells, sequential reparixin therapy could further increase RS with subsequent DNA damage and cell death. However, for the normal cells with low endogenous DNA damage and RS, sequential reparixin therapy may induce minimal increases in RS and consequent DNA damage and cell death.

### Sequential treatment with first-line, second-line chemotherapy, and reparixin inhibits GC growth in vivo

**Fig. 5:**
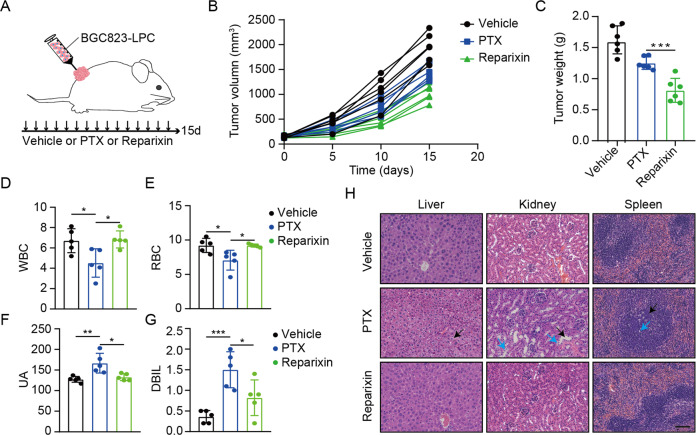
Sequential treatment with first-line, second-line chemotherapy, and reparixin inhibits GC growth in vivo. **A** Schematic representation of the BGC823-LPC cells from tumor tissues subjected to first-line and second-line chemotherapy xenograft model for reparixin treatment. **B** The growth curves of tumor (*n* = 6 per group). **C** The tumor weights were measured and recorded after the tumors were harvested. Data are mean ± standard deviation. ***P < 0.001. **D**, **E** Blood routine index (WBC and RBC) were measured. Data are mean ± standard deviation. *P < 0.05. **F**, **G** Blood biochemical index (DBIL and UA) were measured. Data are mean ± standard deviation. *P < 0.05. **H** The representative images of liver, kidney, and spleen by H&E staining. Scale bars, 50 μm.

To verify the values of sequential therapy targeting IL8 after chemotherapy resistance, a xenograft mouse model was established using primary cells (BGC823-LPC) extracted from tumor tissues, which were subjected to first-line and second-line chemotherapy. The mice were divided into three groups, and were respectively treated with saline, paclitaxel and reparixin (Fig. 5A). The results showed that tumors treated with reparixin were dramatically smaller than those treated with saline or paclitaxel, and reparixin had no significant effect on the body weight of mice (Figs. 5B, C and S7A, B). The efficient knockdown of IL8 was tested by WB assays (Fig. S7C).

Moreover, blood routine examination and blood biochemical analysis showed that reparixin caused no obvious injury to the blood system and solid organs (Fig. 5D–G, Figs. S8 and S9), which was subsequently confirmed by HE-staining (Figs. 5H and S7D). These findings indicated that reparixin could inhibit tumor growth after chemotherapy resistance while minimizing toxicity.

**Fig. 6:**
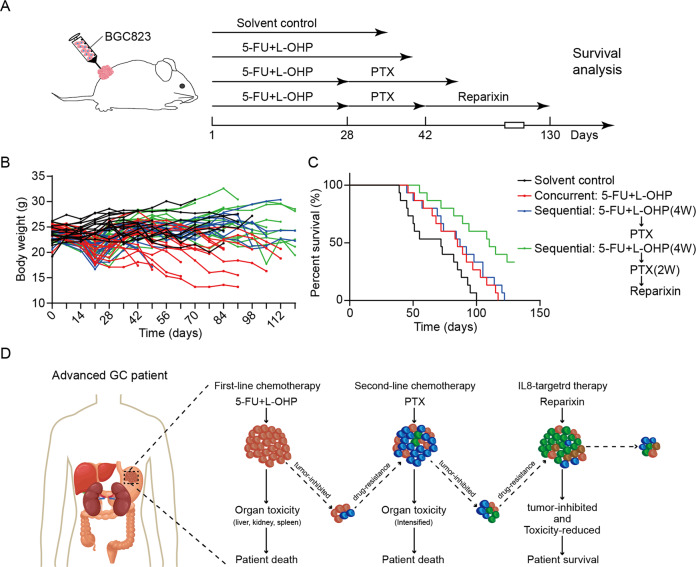
Sequential treatment with first-line, second-line chemotherapy and reparixin prolong the survival time of the GC-bearing mice. **A** Schematic representation of the survival model that subjected to sequential treatment with first-line, second-line chemotherapy and reparixin (*n* = 15 per group). **B** The body weights at the indicated time points after indicated treatments. **C** Kaplan–Meier survival curve after indicated treatments. **D** The graphic illustration of sequential treatment with first-line, second-line chemotherapy and reparixin in suppressing GC growth.

We next conducted sequential treatment of first-line chemotherapy, second-line chemotherapy, and reparixin to observe the effect on prolonging the survival time of mice (Fig. 6A). The results showed that the sequential therapy of 5-Fu+oxaliplatin, paclitaxel, and reparixin effectively prolonged the survival time of the mice compared with the first-line chemotherapy group and sequential chemotherapy group. In addition, the weight of mice were monitored regularly during the survival model to evaluate the side effects of drugs indirectly. The results showed that the weight loss of mice were the most obvious in the first-line chemotherapy group while sequential therapy of 5-Fu+oxaliplatin, paclitaxel, and reparixin did not cause significant weight loss of mice (Fig. 6B, C), suggesting that sequential strategy could reduce accumulation of chemotherapeutic drug toxicity, and reparixin was safe and well-tolerated in vivo.

## Discussion

Tumor foci are composed of cell populations with different genomic characteristics and sensitivity to therapeutic drugs [35]. The difference between these cell populations is an obstacle to cancer therapy. The instability of tumor genome determines that tumor cells may undergo significant mutations after drug therapy, leading to rapid tumor progression or drug resistance [36, 37]. Tumor cells developing heterogeneity during drug treatment are dynamic processes that follow a genetic law [8, 38]. Therefore, analyzing the variation of tumor heterogeneity and finding the key heterogeneous molecules that drive malignant tumor phenotype may be essential for reversing drug resistance. With the rapid development of sequencing technology, we can gain insight into key molecular signals or targets of tumor heterogeneity, while targeting heterogeneity molecular may reverse drug resistance [39, 40]. GC is a molecularly and phenotypically highly heterogeneous disease. One of the major challenges in the current treatment of GC is to translate the latest discoveries in molecular biology into effective treatments for GC patients.

For the initial treatment of patients with metastatic GC, a platinum–fluoropyrimidine doublet is preferred [2]. In the second­line therapy, patients who are fit for chemotherapy might be offered taxane or irinotecan chemotherapy, which has been proved to improve survival compared with best supportive care in randomized trials. However, GC patients have limited benefit from second­-line chemotherapy. The median overall survival of patients treated with second-­line chemotherapy in clinical trials is ~6 months, while the average benefit for overall survival is 6 weeks [41–43]. Chemotherapy resistance has been recognized as a major problem in cancer therapy, reducing the cytotoxic activity of anticancer agents. In addition to drug resistance after repeated use, the obvious toxic effects of chemotherapy drugs limit their clinical application [44]. Hence, the efficacy and tolerance of targeted drugs need to be simultaneously considered when hunting for targets after chemotherapy resistance.

In this study, we used RNA sequencing to screen IL8, a key heterogeneous target that drives drug resistance after chemotherapy in advanced GC. Previous studies have shown that chemotherapy agents induce transcriptional regulation of both the IL8 and IL8 receptor genes, thus increasing the level of autocrine/ paracrine IL8 signaling experienced by cancer cells [23]. It has been found that IL8 derived from CAFs could induce a chemo-resistant phenotype in GC cells in vitro by activating pro-survival PI3K-Akt and NF-κB related signaling networks to withstand the cytotoxic effects exerted by cisplatin therapy [45]. Another study found that the GC cell line MKN45 secreted IL8 as an autocrine growth factor and repertaxin could enhance efficacy of 5-Fu in MKN45 cells [28]. Our study confirmed that IL8 expression was further activated with the increase in drug resistance. For chemotherapy in advanced GC, the activation of IL8 may be the key to drug resistance. Collectively, these results suggest IL8 as a potential marker for predicting chemotherapy resistance in GC patients. In addition, our study confirmed that the overall survival (OS) of GC patients with high IL8 expression was significantly lower than that of GC patients with low IL8 expression, especially in GC patients after 5-Fu therapy. Overexpressed IL8 was obviously associated with tumor size, TNM stage and lymphatic metastasis. It has been reported that IL8 expression impeded recurrence free survival (RFS) in GC patients, indicated by 5-year RFS of 55% and 70% in IL8^High^ and IL8^Low^ patients, respectively [46]. These results suggest IL8 as a potential biomarker for predicting disease progression in GC.

Currently, immunotherapies are transforming outcomes for many GC patients and are quickly becoming the fourth pillar of cancer therapy. The latest version of NCCN guidelines for GC has listed nivolumab combined with fluoropyrimidine and oxaliplatin for tumors with PD-L1 expression levels by CPS of ≥5 for HER2 overexpression negative disease as category 1 preferred options for first-line therapy [47]. Targeting IL8 signaling pathway also exerts broad prospects in immunotherapy of GC. It has been reported that IL8 promoted angiogenesis by recruiting CXCR2^+^ neutrophils, which provided the growing neoplasm with pro-angiogenic mediators (VEGFs and MMPs), implicating IL8 as a druggable immunotherapeutic target [48, 49]. High levels of intratumoural IL8 have been shown to upregulate the expression of PD-L1 on tumor-associated macrophages (TAMs) in GC, corresponding with impaired infiltration and functionality of antitumor CD8^+^ T cells [50]. Moreover, IL8 contributed to tumorigenic inflammation in GC by promoting the influx of neutrophils and abrogating CD8^+^ T cell responses [49, 50], and its receptor CXCR1/2 were involved in the recruitment and activation of myeloid-derived suppressor cells (MDSCs) in the TME [51]. Targeting IL8 signaling pathway remains an attractive therapeutic approach to improve antitumor immunity. However, there were some limits in the Balb/c nude mouse models used in our study, and the effect of targeting IL8 signaling pathway on the immune response in advanced GC remained to be intensively studied.

IL8 signaling pathway is one of the members of chemokine network, and the redundancy of the chemokine system pose a major challenge to implement targeted therapy. Generally, the chemokine receptors are known to recognize more than one type of chemokine. CXCR1 binds CXCL6, CXCL7, and CXCL8 with high affinity, whereas CXCR2 binds to a wide range of chemokines including CXCL1–3 and CXCL5–8 [52]. Therefore, receptor blockade to eliminate the promiscuity of chemokine signaling may be more effective than drugs acting on IL8 alone (including neutralizing antibodies and small interfering RNAs). We selected reparixin, a non-competitive allosteric inhibitor of CXCR1/2, to inhibit IL8 signaling pathway. (2 R)-2-[4-(2-methylpropyl)phenyl]-N-(methylsulfonyl)propanamide(reparixin) was discovered and remolded on the basis of Ibuprofen [53, 54]. It is a potent and selective inhibitor of IL8-induced chemotaxis with a marked selectivity (around 400-fold) for CXCR1 and has been proved efficacious in several ischemia/reperfusion (I/R) experimental models [55]. On the basis of its pharmacological characteristics, reparixin was mainly used for preventing graft rejection after organ transplantation in the early clinical trials. Gradually, researchers have discovered its value in cancer therapy. In this study, reparixin has been shown to suppress xenograft tumor growth, minimize the toxicity and prolong survival in GC mouse models, exerting anticancer activity and safety.

The treatment of patients with advanced GC depends not only on the curative effect, but also on the adverse reactions. Our study found that first-line and second-line chemotherapy caused significant damage to the systemic system of mice and led to toxic death under the restriction of tumor growth. The adverse reactions would be more serious in combined therapy. Sequential therapy strategy could minimize side effects caused by chemotherapy while maintaining efficacy, thus realizing the long-term survival of patients with advanced GC (Fig. 6D). In the present study, we performed sequential therapy with first-line, second-line chemotherapy and reparixin in advanced GC and demonstrated the efficacy and safety of the sequential strategy. Currently, many studies of multi-target sequential interventions for various cancer types have been reported. Combined BET and MEK inhibition resulted in a synergistic decrease in tumor viability in MYCN-expressing triple-negative breast cancer [56]. Combined PD-1, BRAF and MEK inhibition significantly prolonged the survival time of patients with advanced BRAF-mutant melanoma [57]. VEGF inhibitor (Bevacizumab) plus EGFR inhibitor (erlotinib) significantly improved progression-free survival (PFS) in patients with untreated metastatic EGFR-mutated NSCLC [58]. Combined EGFR and FGFR inhibition resulted in meaningful clinical responses in patients with advanced hepatocellular carcinoma [59]. Multitargeted Pan-TRK, ROS1, and ALK Inhibitor Entrectinib demonstrated robust antitumor activity and safety in a broad range of solid tumors [60]. These studies suggest that individualized sequential therapy targeting heterogeneous molecules is a promising strategy to overcome cancer recurrence and achieve long-term survival. In future study, we will follow the principles of efficacy and safety, and focus on targeted drugs that can be combined with reparixin after chemotherapy resistance in advanced GC, so as to provide more appropriate and complete treatment options for GC patients.

Certainly, there were some limits in this study. We did not explore the biology of IL8 system in myeloid leukocyte populations present in immunodeficient mice when using xenografted tumors. Moreover, the modulation of anticancer immune responses by IL8 in vivo remained to be intensively studied. Notably, IL8 is absent from the genome in rodents, thus complicating animal experiments to evaluate in detail the biology of the IL8-CXCR1/2 pathway. Yet, human IL8 is able to act through mouse CXCR1 and CXCR2 allowing xenograft-based experiments [61]. Transgenic mice expressing IL8 can be used to assess the importance of IL8 in a fully immunocompetent mouse in our future study [62]. Furthermore, the potential of reparixin in combination with other targeted drugs and immune checkpoint inhibitors(ICIs) after chemotherapy resistance in advanced GC remained to be explored.

Overall, our study confirms that patients with advanced GC are exposed to the dual threat of chemotherapy resistance and multi-organ toxic effects. By using RNAseq analysis, we accurately identified IL8, a key heterogeneous molecular that led to drug resistance after chemotherapy in advanced GC. Inhibition of IL8 effectively preserved efficacy to GC cells and ameliorated toxicity to normal cells caused by chemotherapy. The sequential treatment with first-line, second-line chemotherapy and reparixin can inhibit GC growth, reduce toxicity and prolong survival. Collectively, our study reveals the potential of targeting IL8 signaling pathway in improving the therapeutic effect of chemotherapy resistance and transforming prognosis, providing new insights for the selection of treatment strategies for patients with advanced GC.

## Materials and methods

### Bioinformatics analysis

The expression pattern of IL8 in GC was obtained from the Gene Expression Profiling Interactive Analysis database [63]. The survival analyses regarding IL8 were performed through the integrated Kaplan–Meier plotter dataset (http://kmplot.com/analysis/).

### GC tissues collection

Tissue specimens were collected from 48 GC patients at the People’s Hospital of Jiangsu Province (Nanjing, Jiangsu, China). Samples of 48 cases were embedded with paraffin to make the tissue microarray, and clinicopathological features, which included age, sex, tumor size, differentiation grade, Lauren classification and TNM stage (American Joint Committee on Cancer classification, AJCC) were shown in Table 1. This study was approved by the Medical Ethics Committee of First Affiliated Hospital of Nanjing Medical University. Written informed consent was obtained from all participants.

### Cell culture and reagents

BGC823, SGC7901 and GES-1 cells were purchased from Type Culture Collection of the Chinese Academy of Sciences (Shanghai, China). All the cell lines were supplemented with 100 μg/ml streptomycin, 100 U/ml penicillin and 10% fetal bovine serum (FBS) at 37 °C in a humidified atmosphere of 5% CO_2_. 5-fluorouracil, oxaliplatin, paclitaxel and reparixin (Selleck Chemicals, USA) were used at the indicated concentrations.

### RNA interference and cell transfection

Small interfering RNAs targeting IL8 were designed and synthesized by RiboBio (Guangzhou, China). The sequences of siRNAs were as follows: si-IL8#1: CTTAGATGTCAGTGCATAA, si-IL8#2: GTCAGTGCATAAAGACATA, si-IL8#3: GCCAAGGAGTGCTAAAGAA. A scrambled siRNA was used as the negative control. The siRNAs were used for transient transfection with Lipofectamine 3000 (Invitrogen, MA, USA).

### RNA extraction and qRT-PCR assays

Total RNA was extracted from tissues and cells with the TRIzol reagent (Invitrogen, MA, USA) according to the manufacturer’s protocol. Isolated RNA was then reversely transcribed in cDNA using HiScript Q RT SuperMix for qPCR (Vazyme, Jiangsu, China). Quantitative RT-PCR was carried out with SYBR Green PCR Master Mix (Vazyme, Jiangsu, China) using an ABI Prism 7900 Sequence detection system (Applied Biosystems, Canada). β-actin was used as an internal control, and the results for each sample were normalized to β-actin expression. The relative quantitative value for each gene was determined as 2^−ΔΔCT^.The primers are listed in Additional file 10.

### RNA-sequencing (RNA-seq) analysis

The total RNA samples were processed by mRNA enrichment method. The mRNA was enriched by using the oligo(dT) magnetic beads. The mRNA was mixed with the fragmentation buffer and broken into short fragments, after which the first strand of cDNA was synthesized by using random hexamer-primer. Buffer, dNTPs, RNase H and DNA polymerase I were added to synthesize the second strand. The double-strand cDNA was purified with magnetic beads. End reparation and 3’-end single nucleotide A (adenine) addition was then performed. Finally, sequencing adaptors were ligated to the fragments. The fragments were enriched by PCR amplification. During the QC step, the Agilent 2100 Bioanaylzer and ABI StepOnePlus Real-Time PCR System were used to qualify and quantify of the sample library, after which the library products were deemed ready for sequencing via Illumina HiSeqTM 2000. The whole RNA-sequencing process and data analysis were conducted by BGI Tech, Shenzhen, China.

### Western blot assays

Total protein was extracted by RIPA lysis buffer supplemented with Protease and Phosphatase Inhibitor. After thorough mixing and incubation at 4 °C for 30 min, lysates were centrifuged at 12,000 × *g* at 4 °C for 15 min, and supernatants were collected. The protein content of lysates was determined, and lysates were separated by 10% SDS-PAGE, and electro-transferred onto polyvinylidene difluoride (PVDF) membranes. After blocking with 5% non-fat milk in TBST for 1 h, membranes were incubated with primary antibodies at 4 °C overnight, followed by horseradish peroxidase (HRP)-conjugated secondary antibodies (Beyotime, Shanghai, China) at room temperature for 1 h. Bands were visualized using BeyoECL Star kit (Beyotime, Shanghai, China). Tubulin was regarded as the loading control. The expression of target protein is equal to the gray value of target protein bands divided by the gray value of Tubulin. Primary antibodies were as follows: anti-IL8 (Abcam, ab106350); anti-cleaved PARP (Cell Signaling Technology, #9542); anti-Bcl2 (Proteintech,12789-1-AP); anti-Bax (Proteintech, 50599-2-Ig); anti-α-tubulin (Beyotime, AF0001).

### Cell counting kit-8 (CCK8) assay

Cell survival and proliferation were assessed by Cell Counting Kit 8 (NCM Biotech, Jiangsu, China). Cells were seeded into 96-well plates overnight and subsequently treated with indicated drugs for 48 h. Ten microliters of CCK8 solution was added to each well, followed by incubation for 1–2 h at 37 °C. The absorbance at 450 nm was measured using an automatic microplate reader (Synergy4; BioTek, Winooski, VT, USA). In addition, after 48 h of transfection, GC cells (3 × 10^3^) were seeded into 96-well plates, and cell viability was recorded every 24 h.

### Colony formation assay

Cells were seeded in six-well plates and allowed to adhere overnight and treated in accordance with the experimental design. Media and drugs were changed every 3 days. After 21 days, remaining cells were fixed with methanol and stained with 0.1% crystal violet. In addition, after 48 h of transfection, GC cells were seeded into six-well plates, and colonies were measured after 10 days.

### 5-Ethynyl-2′-deoxyuridine (EdU) incorporation assay

EdU assays were performed with a Cell-Light EdU DNA Cell Proliferation Kit (RiboBio, Guangzhou, China). Cells were seeded 50% confluent in 96-well plates after 48 h of transfection and were continuously cultured for 24 h. After the incubation with 50 μM EdU for 2 h, cells were fixed in 4% paraformaldehyde, followed by the permeabilization in 0.5% Triton X-100 diluted in PBS. Then the cells were stained with Apollo Dye Solution. Hoechst 33342 was used to stain the nucleic acids within the cells. Images were obtained with a Nikon Ti microscope (Nikon, Tokyo, Japan), and the number of EdU-positive cells was counted.

### Transwell assay

Transwell migration assay were performed in 24-well plates, using a 6.5-mm diameter Transwell chamber with 8-μm pore polycarbonate membrane insert (Corning, MA, USA). After 48 h of transfection, GC cells (3 × 10^4^) were plated on the upper chambers in serum-free medium. RPMI 1640 containing 10% FBS was added to the lower chambers as a chemoattractant. After incubation for 24 h at 37 °C, cells were fixed with methanol, stained with crystal violet solution, and counted using an inverted microscope. The numbers of cells counted in five random fields were averaged.

### Flow cytometry assay

For apoptosis assays, the harvested cells were stained with PI and Annexin V-FITC according to the manufacturer’s instructions (Vazyme, Jiangsu, China). For cell cycle assays, the harvested cells were fixed overnight in 70% ethanol at 4 °C. Cells were incubated with PI staining solution (BD Biosciences) before flow cytometry detection. The percentage of cells in the phase of G0/G1, S or G2/M was calculated and compared according to FACScan analysis.

### Immunofluorescence

Cells were seeded into a confocal dish and treated in accordance with the experimental design. The cells were fixed in 4% paraformaldehyde for 15 min and then permeabilized with 0.25% Triton X-100 at room temperature. Next, the cells were blocked with 1% bovine serum albumin for 1 h and incubated with primary antibody targeted to γ-H2AX (Abcam, ab81299) at 4 °C overnight. After washing with TBST three times, the cells were incubated with anti-rabbit Alexa fluor-488-conjugated secondary antibody (Proteintech, SA00013-2) for 1 h at room temperature. The nuclei were stained with 4′, 6-diamidino-2-phenylindole (DAPI), and images were observed with an Eclipse fluorescence microscope (Nikon, Tokyo, Japan).

### Comet assay

A comet assay was performed using a DNA Damage Detection Kit (Keygen, China). The cells were treated in accordance with the experimental design. Cells were harvested and suspended in PBS containing 0.7% low-melting agarose and layered onto adhesive microscope slides previously covered with 1% normal-melting agarose. The cells were dipped in a specific lysed buffer at 4 °C for 1–2 h. Next, the DNA was uncoiled and unwound in an alkalescent electrophoresis buffer for 30 min. Electrophoresis was performed at 25 V for 30 min. After neutralization with 0.4 mM Tris-HCl buffer (pH 7.5) for three times, the cells were stained with PI solution for 10 min in a dark room. The slides were examined with an Eclipse fluorescence microscope (Nikon, Tokyo, Japan).

### Immunohistochemical (IHC) analysis

For hematoxylin-eosin stain (HE) staining, human or xenograft tumors were embedded and sectioned, and then stained with hematoxylin and eosin. For immunohistochemical studies, the samples were incubated with primary antibody against IL8 overnight at 4 °C. After three successive rinses with a washing buffer, the second antibody was added for 60 min at room temperature. The signal was amplified and visualized with 3′-diaminobenzidine chromogen (DAB), and then counterstained with hematoxylin.

### Mouse models

All animal experiments were performed in accordance with a protocol approved by the Institutional Animal Care and Use Committee of Nanjing Medical University. BALB/c nude mice (male, 4–6 weeks old) were obtained from SLAC Laboratory Animal Center (Shanghai, China) and maintained in SPF facilities. The mice experiments were blind.

To establish a chemotherapeutic drug-resistance model, 5 × 10^6^/100 μL of BGC823 cells were subcutaneously injected into the right axilla of BALB/c nude mice. When the tumor volume (volume = length × width^2^/2) reached about 100 mm^3^, the mice were randomly divided into two groups: control (*n* = 8) and 5-Fu plus oxaliplatin (*n* = 40). Saline, 5-Fu (20 mg/kg) and oxaliplatin (5 mg/kg) were injected intraperitoneally every 3 days. When the tumor volume reached about 1500 mm^3^, 16 mice (8 mice from the control group and 8 mice from the chemotherapy group) were sacrificed and the tumors were weighed and imaged and then frozen for further analyses. The remaining mice were treated with 5-Fu and oxaliplatin until developing drug resistance. Eight mice from the first-line chemotherapy resistance group were sacrificed and the tumors were frozen for further analyses.

Next, the mice were randomly divided into two groups according to the volume of xenograft tumors (*n* = 12 per group): one group was still treated with 5-Fu plus oxaliplatin and one group was treated with paclitaxel (10 mg/kg) twice a week. During the experiment, the natural death time of mice was recorded. The endpoint of the experimental therapy was 2 weeks after paclitaxel treatment. The mice were sacrificed, and the tumors were dissected and then frozen for further analyses.

To verify the effectiveness of targeting IL8 after first-line and second-line chemotherapy resistance, mouse models were established to examine the effects of reparixin sequential therapy on tumor proliferation and mouse survival. Primary cells (BGC823-LPC) extracted from tumor tissues that were subjected to first-line and second-line chemotherapy were injected into the right axilla of BALB/c nude mice. Then the mice were randomly divided into three groups (*n* = 6 per group) and treated with (1) saline; (2) paclitaxel (10 mg/kg) twice a week; reparixin (30 mg/kg) every day for 15 days. The mice were sacrificed, and the tumors were weighed and frozen for further analyses.

Additionally, the BALB/c nude mice were injected with 5 × 10^6^ BGC823 cells in 0.1 ml of PBS into the right axilla. Next, the mice were randomly divided into four groups (*n* = 15 per group) and treated with (1) saline; (2) 5-Fu (20 mg/kg) and oxaliplatin (5 mg/kg) every 3 days; (3) 5-Fu (20 mg/kg) and oxaliplatin (5 mg/kg) every 3 days for 4 weeks and paclitaxel (10 mg/kg) twice a week;(4) 5-Fu (20 mg/kg) and oxaliplatin (5 mg/kg) every 3 days for 4 weeks, paclitaxel (10 mg/kg) twice a week for 2 weeks and reparixin (30 mg/kg) every day until the mice die. The death time of mice was recorded.

### Statistical analysis

All analyses and depicted graphs were performed with GraphPad Prism 8. Data were presented as the means ± standard deviation (SD). The student’s *t*-test was used to analyze differences between groups, while one-way ANOVA was used for analyzing more than two groups. Statistical significance was defined as a *P-*value less than 0.05. (*P-value < 0.05; **P < 0.01; ***P < 0.001; ns, no significance).

## Supplementary information

Supplementary figures and tables

## Data Availability

The datasets used and/or analyzed during the current study are available from the corresponding author upon reasonable request.
