# Supplementary material for: Targeting IL8 as a sequential therapy strategy to overcome chemotherapy resistance in advanced gastric cancer
Source: Cell Death Discov. 2022 Apr 29;8:235. doi: 10.1038/s41420-022-01033-1 (PMC9055054; doi:10.1038/s41420-022-01033-1)
Supplement: Supplementary file 1 — Supplementary figures and tables [file 41420_2022_1033_MOESM1_ESM.pdf]

Targeting IL8 as a sequential therapy strategy to overcome chemotherapy resistance in advanced gastric cancer

Huning Jiang[1†], Jiahua Cui[1†], Hao Chu[1], Tingting Xu[1], Mengyan Xie[1], Xinming Jing[1], Jiali Xu[1], Jianwei Zhou[2,3], and Yongqian Shu[1,2,4*]

Supplementary figures and tables

Additional file 1: Fig. S1. First-line chemotherapy effectively inhibits GC growth but develops drug resistance.

Additional file 2: Fig. S2. Second-line chemotherapy reverses resistance to first-line chemotherapy of GC.

Additional file 3: Fig. S3. Blood routine assays of mice after sequential chemotherapy.

Additional file 4: Fig. S4. Blood biochemical assays of mice after sequential chemotherapy.

Additional file 5: Fig. S5. Screening IL8 as the therapeutic target after chemotherapy resistance.

Additional file 6: Fig. S6. The suppression of IL8 attenuated the proliferation and migratory capacity of SGC7901 cells.

Additional file 7: Fig. S7. Sequential treatment with first-line, second-line chemotherapy and reparixin inhibits GC growth in vivo.

Additional file 8: Fig. S8. Blood routine assays of mice treated with reparixin.

Additional file 9: Fig. S9. Blood biochemical assays of mice treated with reparixin.

Additional file 10: Primers for qRT-PCR

Additional file 11: Table S1. DEGs in enriched signaling pathways after sequential chemotherapy resistance in advanced GC.

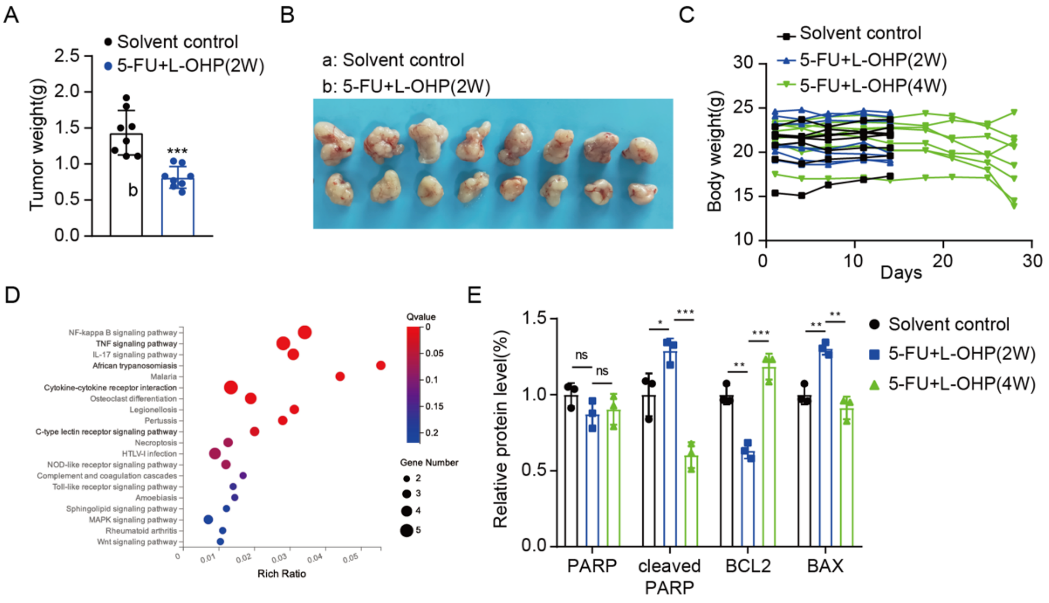

Additional file 1: Fig. S1. First-line chemotherapy effectively inhibits GC growth but develops drug resistance. (A) The tumor weights were measured and recorded after the tumors were harvested (n=8). Data are mean ± standard deviation. ***P < 0.001. (B) The images of dissected tumors from BGC823 treated with saline or 5-Fu plus oxaliplatin (n=8). (C) The body weight curves at the indicated time points after indicated treatments (n=8). (D) KEGG analysis for altered genes after four weeks compared with two weeks of first-line chemotherapy. (E) The relative protein expression of apoptosis-related genes (PARP, cleaved PARP, Bcl2, and Bax) were measured by ImageJ (n=3). Data are mean ± standard deviation. *P < 0.05, **P < 0.01, ***P < 0.001.

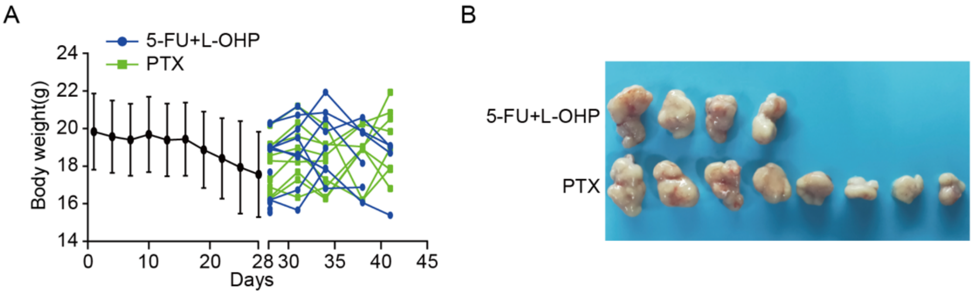

Additional file 2: Fig. S2. Second-line chemotherapy reverses resistance to first-line chemotherapy of GC. (A) The body weight curves at the indicated time points after indicated treatments (n=12). (B) The images of dissected tumors at the end of the model. Four and eight mice survived in the 5-Fu + oxaliplatin group and paclitaxel group respectively.

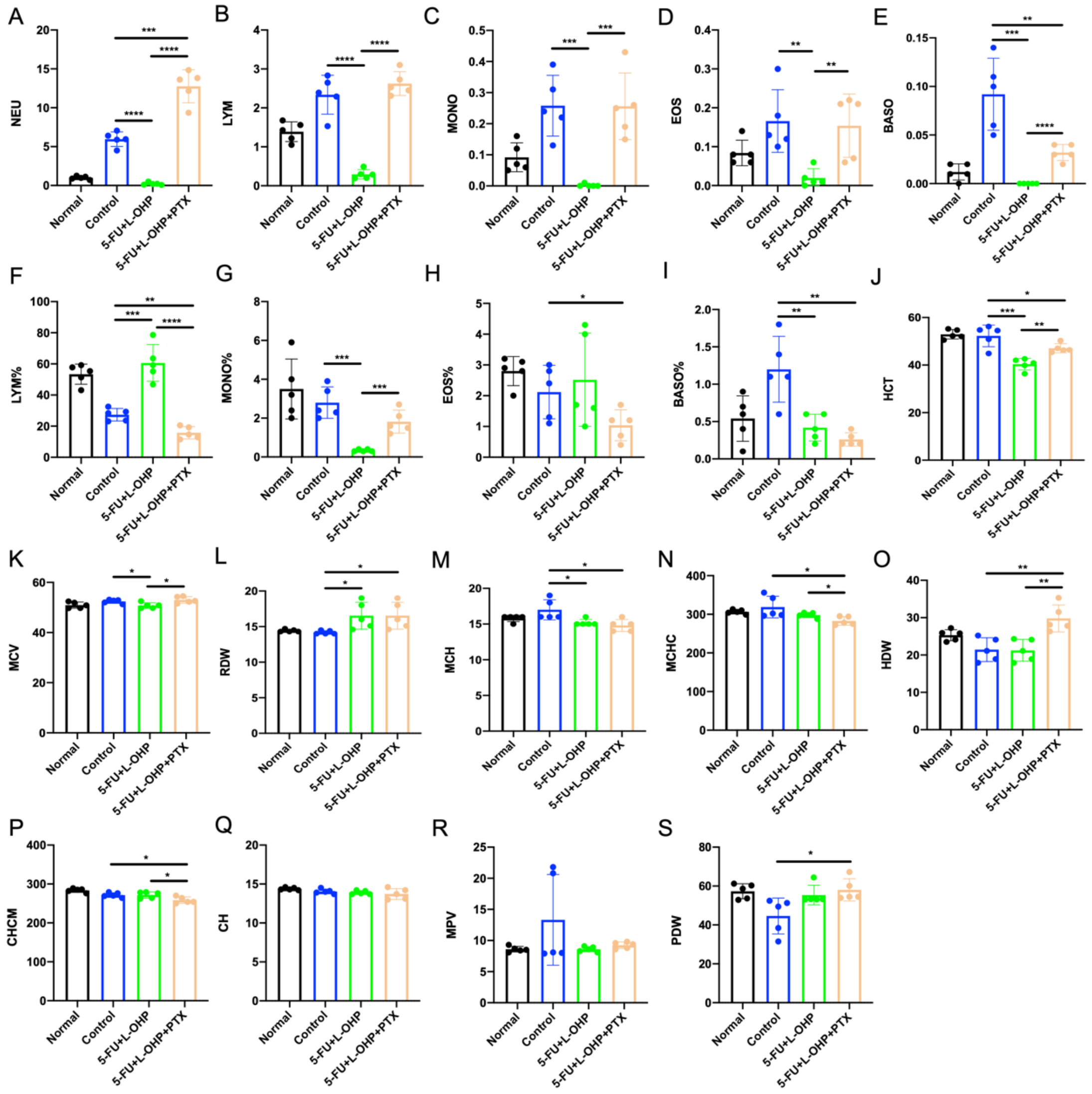

Additional file 3: Fig. S3. Blood routine assays of mice after sequential chemotherapy (n=5).

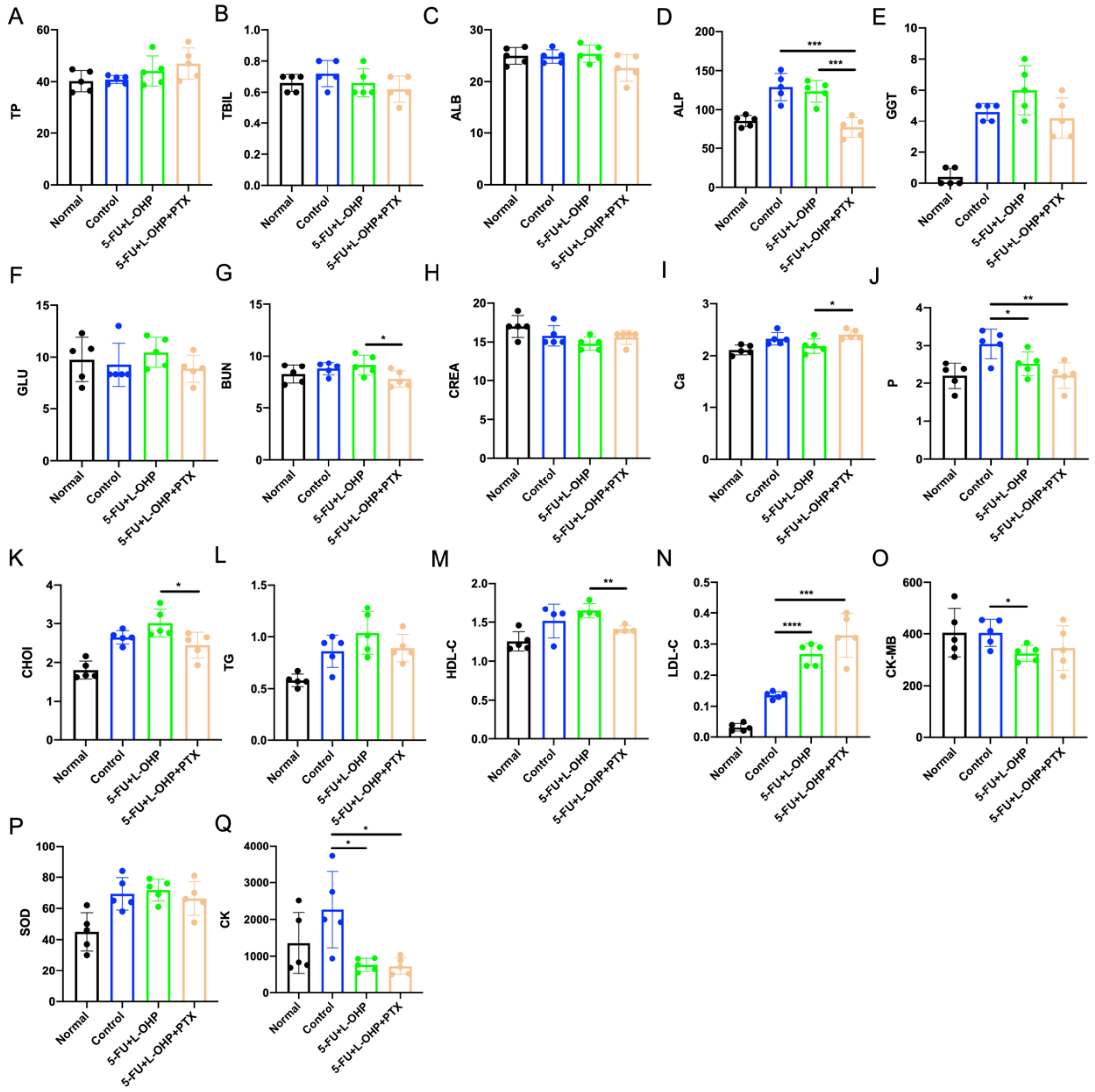

Additional file 4: Fig. S4. Blood biochemical assays of mice after sequential chemotherapy (n=5).

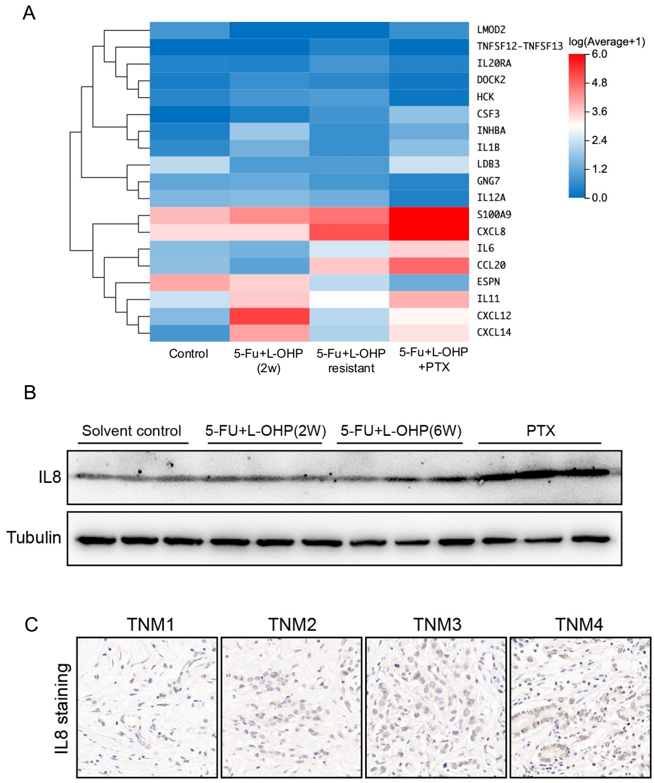

Additional file 5: Fig. S5. Screening IL8 as the therapeutic target after chemotherapy resistance. (A) Heatmap of DEGs in the three enriched signaling pathways most associated with cancer at different stages of chemotherapy. (B) IL8 expression at different periods of chemotherapy was detected by western blot analysis (n=3). (C) IL-8 expression was positively correlated with TNM stage as shown by IHC staining.

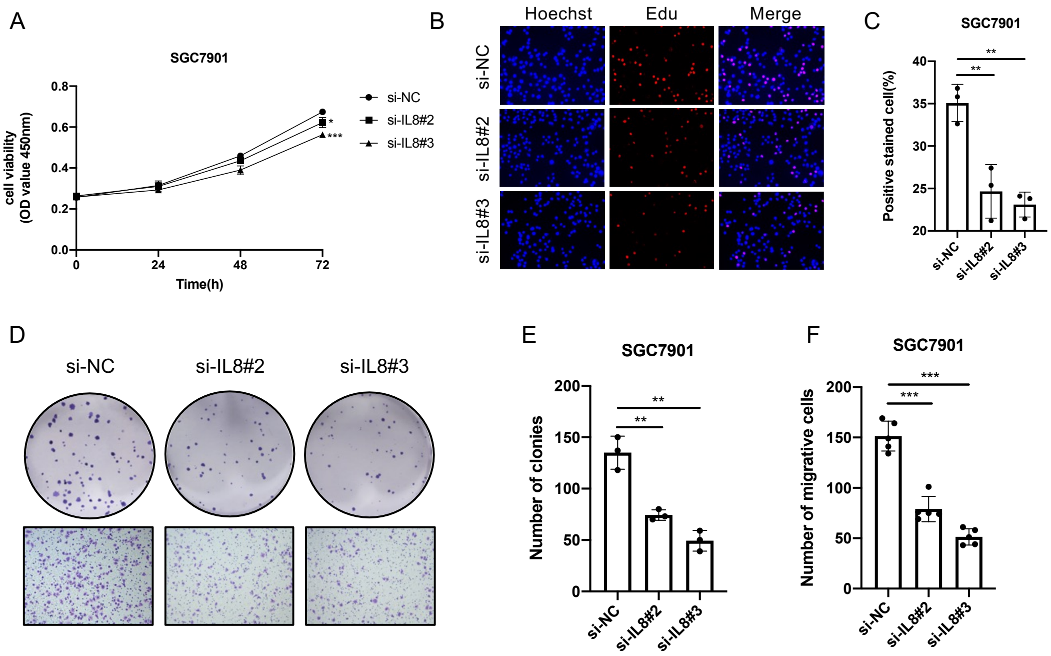

Additional file 6: Fig. S6. The suppression of IL8 attenuated the proliferation and migratory capacity of SGC7901 cells. (A) CCK8 assays was carried out to detect the viability of SGC7901 cells transfected with si-IL8#2 and si-IL8#3. *P < 0.05, ***P < 0.001. (B-C) Representative images (B) and quantification (C) of SGC7901 cells transfected with si-IL8#2 and si-IL8#3 by EdU staining assays. Data are mean ± standard deviation. **P < 0.01. (D-F) Representative images (D) and quantification (E-F) of SGC7901 cells transfected with si-IL8#2 and si-IL8#3 by colony-forming experiments and transwell assays. Data are mean ± standard deviation. **P < 0.01, ***P < 0.001.

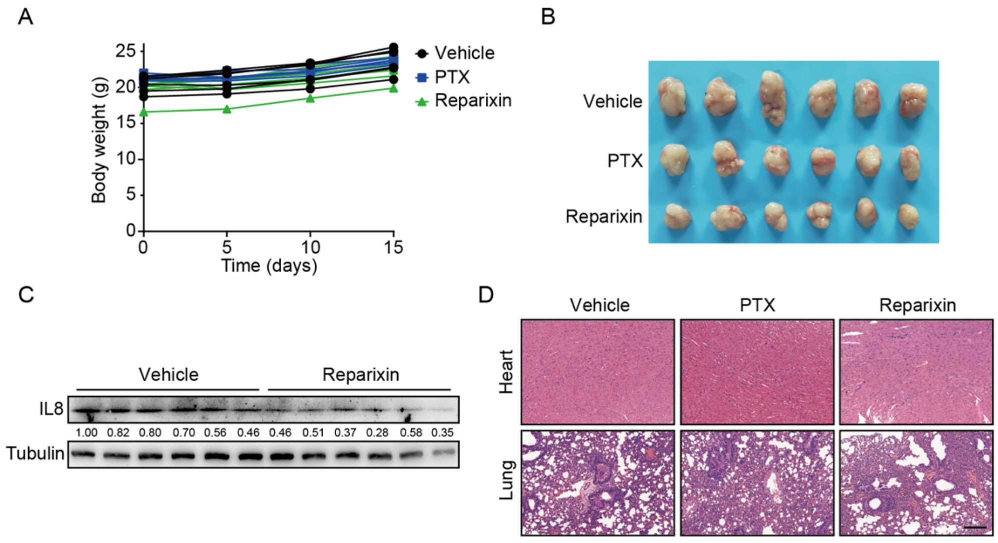

Additional file 7: Fig. S7. Sequential treatment with first-line, second-line chemotherapy and reparixin inhibits GC growth in vivo. (A) The body weight curves at the indicated time points after indicated treatments (n=6). (B) The images of dissected tumors from BGC823 treated with saline, paclitaxel or reparixin (n=6). (C) IL-8 expression in dissected tumor tissues treated with saline or reparixin was detected by western blot (n=6). (D) The representative images of heart and lung by H&E staining. Scale bars, 100 μm.

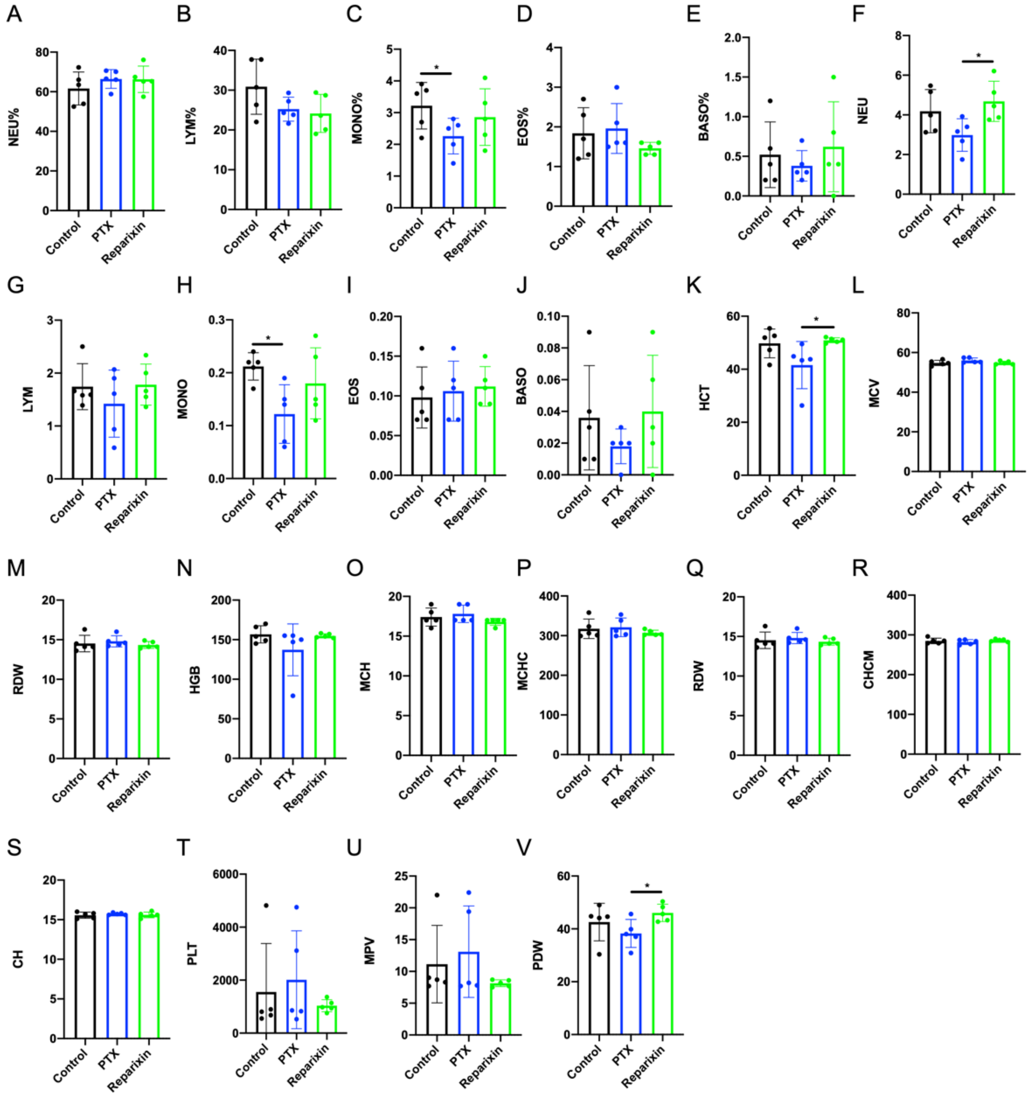

Additional file 8: Fig. S8. Blood routine assays of mice treated with reparixin (n=5).

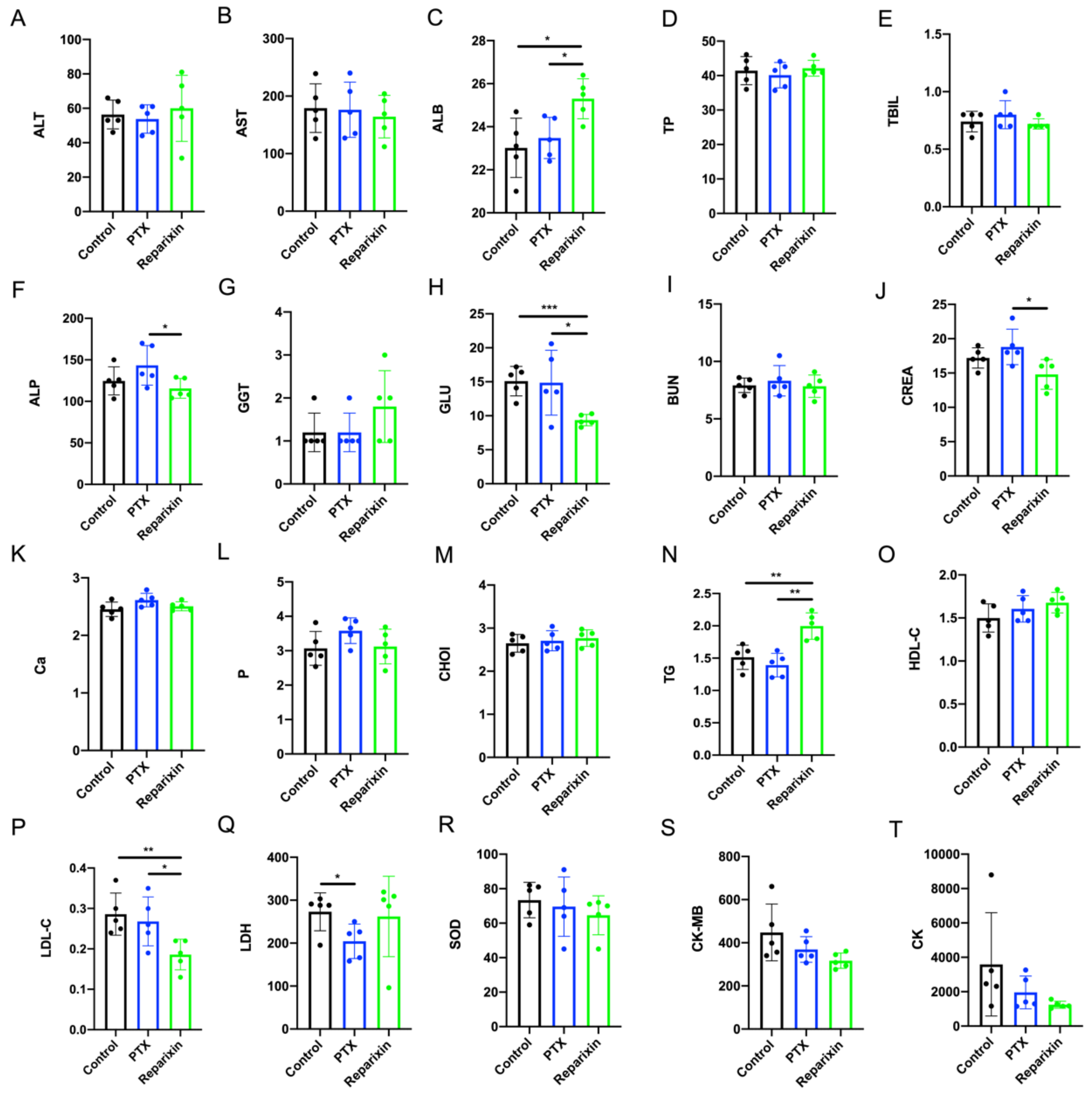

Additional file 9: Fig. S9. Blood biochemical assays of mice treated with reparixin (n=5).

Additional file 10: Primers for qRT-PCR

LDB3-F: TCCAAGCGTCCTATCCCCATC

LDB3-R: TGTATTCTGTCCCGGTCATCTG

CSF3-F: GAGCTTCCTGGAGCTGGCGTAC

CSF3-R: TGCTACAGGCGGGAGAAT

IL1B-F: AGCAACAAGTGGTGTTCTCC

IL1B-R: TGGGATCTACACTCTCCAGC

IL6-F: GCCAGAGCTGTGCAGATGAG

IL6-R: TGTCATGTCCTGCAGCCACT

CXCL8-F: GACATACTCCAAACCTTTCC

CXCL8-R: AACTTCTCCACAACCCTCTG

IL11-F: CTCATCGCTGCTCCTCT

IL11-R: GCTTGCGTGTTGTTCTG

IL12A-F: ACGAGAGTTGCCTGGCTACTAG

IL12A-R: CCTCATAGATGCTACCAAGGCAC

INHBA-F: GCCCCTTTGCCAACCTTAA

INHBA-R: ATGGACATGGGTCTCAGCTT

TNFSF12-F: GGCCTCGAAGAAGTGTTTCTAA

TNFSF12-R: GGAGCTGTTGATTTTGGTCTCT

TNFSF13-F: CCTTGCTACCCCACTCTTG

TNFSF13-R: ACACTCAGAATATCCCCTTGG

IL20RA-F: GCTGCTGCTGTTGCTCCTG

IL20RA-R: CAGTGTAAGTAACTTTAACTCC

CCL20-F: CCTCTGCGGCGAATCAGAAG

CCL20-R: CTGCCGTGTGAAGCCCACAA

CXCL12-F: GGAGGATAGATGTGCTCTGGAAC

CXCL12-R: AGTGAGGATGGAGACCGTGGTG

CXCL14-F: AGCACTGCCTGCACCCTAAG

CXCL14-R: TCTCGTTCCAGGCATTATACCA

S100A9-F: CATGGAGGACCTGGACACAAA

S100A9-R: CTCGTGCATCTTCTCGTGGG

DOCK2-F: CTTGGAGG TCCTCAGCTGTC

DOCK2-R: GTCTGAGCTGGTCTGGAAGG

HCK-F: AGCCTGCTGGACTTCCTGAAGAG

HCK-R: CTCGGAGGTCTCGGTGGATGTAG

LMOD2-F: AGAGAAAACCCCCACAGGGA

LMOD2-R: TTGTCTTCTGCAACCTTTCCA

BAX-F: GCGACTGATGTCCCTGTCT

BAX-R: TGAGTGAGGCGGTGAGC

BCL2-F: GCGGATTGACATTTCTGTG

BCL2-R: CATAAGGCAACGATCCCA

KI67-F: TATGCCTGTGGAGTGGAAT

KI67-R: GGGTGAGAAAAGGTGCTG

β-actin-F: AGGCACCAGGGCGTGAT

β-actin-R: GCCCACATAGGAATCCTTCTGAC

Additional file 11: Table S1. DEGs in enriched signaling pathways after sequential chemotherapy resistance in advanced GC.

Enriched signaling pathways	DEGs
Cytokine-cytokine receptor interaction	LDB3，CSF3，IL1B，IL6，**IL8**，IL11, IL12A，INHBA，TNFSF12-TNFSF13，IL20RA，**CCL20**，CXCL12，ESPN，CXCL14
IL-17 signaling pathway	CSF3，IL1B，IL6，**IL8**，S100A9，**CCL20**
Chemokine signaling pathway	DOCK2，GNG7，HCK，**IL8**，LMOD2，**CCL20**，CXCL12，CXCL14
